# Urothelial Carcinoma

**DOI:** 10.1155/2012/461370

**Published:** 2012-09-11

**Authors:** Nan-Haw Chow, Margaret Knowles, Trinity J. Bivalacqua

**Affiliations:** ^1^Department of Pathology, College of Medicine, National Cheng Kung University, No.1 University Road, Tainan City 701, Taiwan; ^2^Leeds Institute for Molecular Medicine, Section of Experimental Oncology, Cancer Research UK Clinical Centre, St Jame's University Hospital, Beckett Street, Leeds LS9 7TF, UK; ^3^Johns Hopkins Medical Institutions, The James Buchanan Brady Urological Institute, 600 N. Wolfe Street, Marburg 409, Baltimore, MD 21287, USA

Bladder cancer is a common urologic cancer. In North America, South America, Europe, and Asia, the most common type of epithelial tumor diagnosed is urothelial (transitional cell) carcinoma. Worldwide, however, squamous cell carcinoma is the most common form of bladder cancer, accounting for 75% of cases in developing nations. Clinical management of this type of bladder cancer is challenging due to the heterogeneity in bladder tumors with respect to invasion and metastasis and frequent recurrence in the bladder among patients treated with bladder preservation therapies. These dilemmas of clinical practice have stimulated translational research. At the same time, the characteristics of environment-driven carcinogenesis and divergent molecular pathways in the development of low- and high-grade tumors provide a unique opportunity for molecular research in cancer biology. Bladder cancer is also at the forefront of biomarker development because of the ease of developing noninvasive urine tests.

This special issue presents the up-to-date summaries related to molecular pathogenesis, diagnosis, prognostic assessment, and management of bladder cancer. The editorial board selected 8 papers that address these special research areas in the urothelial carcinoma. In the basic research section, Han et al. describe the methylation pattern of noninvasive and invasive bladder cancers, respectively. It appears that two epigenetic pathways give rise to two tumor types, and certain epigenetic alterations precede histopathological changes. The dynamic nature and reversibility with pharmacological interventions make it an excellent target for epigenetic therapy in the future.

Martino et al. provide a comprehensive review of the molecular alterations of fibroblast growth factors receptors in urothelial carcinogenesis. They underscore the potential of the receptors and downstream signaling pathways as therapeutic targets, diagnostic and prognostic markers, and screening tools for early detection and clinical management of urothelial cancer.

Another novel treatment for urothelial carcinoma is described by K. G. Potts et al. They review recent advancements of therapy using oncolytic viruses engineered to selectively replicate in and lyse tumor cells leaving normal cells unharmed. Although encouraging safety profiles and antitumor activity have been demonstrated with a variety of oncolytic viruses, the ultimate proof still needs to be provided by randomized Phase III clinical trials.

 With respect to molecular diagnosis of bladder cancer, Reinert et al. discuss the currently available biomarkers NMP22, ImmunoCyt, and UroVysion. All of three markers have a higher sensitivity than cytology, but a lower specificity. This review focuses on the urinary DNA methylation markers in the diagnosis and surveillance of bladder cancer.

 With respect to medical treatment, Askeland et al. summarize the updated progress of immunotherapy for nonmuscle invasive bladder cancer. The agents include INF-*α*, IL-2, IL-12 and IL-10, either as adjuncts with *Mycobacterium bovis *bacillus Calmette-Guérin treatment or as a solo replacement therapy.

Tsai et al. report a meta-analysis to examine the evidence related to the predictive importance of ErbB receptor signaling in bladder cancer. They demonstrate that the overall risks of disease progression for patients with EGFR or ErbB2 overexpression are 4.5 and 1.1, and the risks of mortality are 3.0 and 1.1, respectively. Future direction of marker assessment should focus on the significance of coexpression patterns of the ErbB receptor family.

Mohamed et al. discuss the impact of different treatment options for muscle invasive bladder cancer patients on the quality of life in the short and long term, particularly concerning urinary diversion. They also call attention to the challenges that patients and family caregivers may face, including psychological, physical and social daily living, emotional support, and interpersonal communication.

A paper contributed by Hyndman et al. presents the novel technology for neo-urinary conduit seeded with autologous smooth muscle cells. This construct may potentially eliminate the complications associated with the use of gastrointestinal segments in urinary reconstruction and greatly facilitate recovery from cystectomy.

 It is hoped that this special issue can bring the latest information to all urologists, pathologists, and medical oncologists who wish to provide their patients with the most comprehensive care.

*Nan-Haw Chow* *Margaret Knowles* *Trinity J. Bivalacqua*

